# Maryland State Dental Association

**Published:** 1892-05

**Authors:** 


AMERICAN JOURNAL

—OF—

DENTAL SCIENCE.

VOL. XXVI. BALTIMORE, MAY, 1892. NO. I.

ARTICLE I.—ORIGINAL.

### MARYLAND STATE DENTAL ASSOCIATION.

The annual session was held May 9 and 10, 1892, in St. James' Hotel, Baltimore; Cyrus M. Gingrich, D.D.S., President, in the chair; W. W. Dunbracco, D.D.S,, Secretary.

### PRESIDENT GINGRICH'S ADDRESS.

*Gentlemen of the Maryland State Dental Association:*

As it is the custom that the presiding officer of our Association deliver an address, in conformity thereto only a few moments of your time will be occupied making a few suggestions for your consideration.

Baltimore is the birth-place of dentistry. The star of our profession first shed its light and transcendent glory to the world from the city in which we are assembled to-night. Being upon our native soil in the "land of our fathers," nothing could be more appropriate than to take a retrospective view of the achievements accomplished, but time will not permit even a glance at the past. It will be enough to say that in the short space of fifty years dentistry has sent glad tidings to every nation upon the earth, and "where'er the sun doth his successive journeys run," we offer a "healing balm" to suffering humanity.

No influence has been so potent and so powerful in advancing our profession, and nothing has given us a greater impetus to place us upon the high plane upon which we are standing to-day, than dental associations. Our dental colleges have done a great work, and are primarily the natural starting place for the student, but our societies have been as a post-graduate course to the individual, and a *special* inspiration to the practitioner, where he meets his professional brother face to face, to engage in the higher spheres of professional attainments. The profession recognized the *truth* when urging dental colleges to extend their scholastic term three years, before granting diplomas to students, that our hope for the future must be in a higher educational standard. We are moving in an age of light and knowledge, an age in which science and art are marching onward, and whatever useful and engaging endowments we possess, we must wield with unflinching energy,—to stand still is to retrograde. What we can effect as a profession depends on what we are. If our work is small and narrow, we will have no force, no weight, no influence in the community. If we push onward and forward "still achieving still pursuing," our work will be massive, our influence cogent, the power of our profession will be felt as a "kingly spirit having authority."

The philosophical and energetic student will find in the realm of dentistry a field wide enough, and comprehensive enough, to engage the most liberal mind in the acquisition of knowledge and truth which alone creates a scientific spirit and comprehension of natural laws of order and of necessity. Of this necessity, dentistry was born, and thus brings us face to face with the ground work upon which our profession must ever stand; upon this basis the men of culture must build, and by patient application and investigation they will be brought to perceive the unity of histology, anatomy and physiology; discover under biology, chemistry—under chemistry, physics—under physics, mechanics and mathematics. From the stand-point thus arrived at, the scientific dentist reasons, analyzes, compares, weighs and measures, and is thus brought to the logical phenomenon of cause and effect.

To simply affirm that a tooth has decayed, and that a gold filling inserted into the cavity will preserve it, will not satisfy an inquiring mind. The mind must have an ultimate fact upon which to repose. It inquires for the cause of the decay. It is not sufficient to tell it; that is a law of nature. This it regards as no cause, for law is only a mode of action, and is therefore of itself nothing. This may satisfy a school-boy for a time, but a philosophic mind regards the assignment of the cause as but a restatement of the phenomenon in question. It cannot regard the mere fact as the end of all philosophic inquiry, neither can it rest in an eternal succession of causes. It *demands* a *first* cause; credulous ignorance would very probably accept the condition as presented as final, with no care or thought as to cause, but not so with the learned man—he *must* reason.

The mind sees material things exist, and it demands their origin and a recognitory basis for their being. So to return to the first proposition we will see by reflection as we already know by experience, that we are brought face to face with the powerful reality that "*knowledge is power*" and alone emancipates the reason, because it dissipates ignorance and superstitions of every kind, and teaches the *true*, the possible and the useful. In brief it is the only elevator of the professional mind, and only opens to the soul large horizons, capable of comprehending the scientific unity and ground work of all that is implied in the profession and practice of dentistry.

The Maryland State Dental Association is reminded there is yet a great work to be done to place the profession where it properly belongs. We have done very little in Maryland to add to the material interests and elevation of the profession at large. If we wish to join the march of fame, we must "be up and doing." "The profession calls for champions, and the fields are white unto the harvest." There is not one man in the society who has not the capacity to accomplish at least one useful and important worthy purpose, for the general advancement of dentistry. It will require *purpose*, *will* and *oneness* of aim, and invincible determination to meet all the demands, hopes, aims and prospects.

We invite the young men of Maryland to join the State Association and become identified with the profession. Young men and young workers are wanted. There are prizes enough for every earnest and faithful worker and crowns enough for every honorable *head* that is willing to go through the smoke of conflict to victory.

We say to the young men, there will be obstacles to be surmounted and difficulties to be vanquished, yet with truth and integrity for your watch-word, and leaning on your own noble purposes and indefatigable exertions, you will be honored by your profession, and be an ornament to your community.

Much has been said about the erection of monuments and memorials to the memory of Drs. Harris and Hayden and their faithful co-laborers, who devoted the best efforts of their lives to the profession of dentistry, in establishing the fundamental principles upon which we to-day stand. Their names are written imperishably upon the pages of dental history, and in view of the reverence and grateful remembrance of them, what more endearing and grateful memorial could be dedicated to their memory, than the Maryland State Dental Association, by infusing new life and energy, to make it the central attraction for the dental profession of this country. We would only be perpetuating the work so grandly begun by them.

We remind the association that at our last annual meeting out of seven committees appointed by the President, only two submitted reports. When committees are appointed and called upon to render reports to the society, excuses, however plausible, avail nothing—neither can they be accepted. It should be considered a trust of honor, and faithfully performed. When committees fail to render reports the primary objects of the society are lost. The success of the society does not depend upon the President, but upon the united co-operation of each individual member.

In order to give this society additional strength and interest, it has been suggested that we invite the society of the District of Columbia to meet with us, and meet alternately in Baltimore and Washington. If such an arrangement could be effected amicably, much strength could be added to the annual meetings.

"Death loves a shining mark," and since we last met the destroyer has had a rich harvest from our ranks. Many of our most distinguished brethren have been called hence, and it is hoped the Association will appoint a special committee to draft resolutions suitable to their memory, to be placed upon the records of the society.

### DENTAL LEGISLATION.

BY H. B. NOBLE, D. D. S , OF WASHINGTON.

Thirty-nine states have dental laws. Most of them have been passed as they now stand, within the last half dozen years; many of them, we think, are not in accord with the best thought of the profession and will, like all young vines, need careful pruning and training, to produce the best and most desirable fruit.

Most of them give too much power to examining boards—a power they have not been slow to use before even their own fitness for the position had been tested or proved. A seeming antagonism between state boards and colleges has appeared. That was unnatural, and if persisted in, will be hurtful to our dental colleges, which no thoughtful lover of dental education can approve.

The work of our dental colleges is the foundation upon which to build a scientific, capable dentist, and all action of state examining boards, or of dental legislation that tends directly or indirectly to bring into disrepute the diplomas of reputable dental colleges, or of college training and work is to be deplored.

College work and diplomas may not in all cases be what they should be; but will the discarding of diplomas by examining boards tend to more careful work in our colleges? We think not. If examinations are conducted, as in some of the states, making no distinction between the good and the poor, will it not be detrimental to dental colleges? Are the men on the examining boards as a rule qualified to sit in judgment on the college professor's work, or even to ascertain the knowledge imparted or possessed by the student, and understood by him? It requires training and practical experience to ascertain just what a student knows. The teacher by daily contact for months with a student can best determine what he knows or is capable of doing.

The state boards ought to visit the colleges in their states, and a representative at least be present at the examination in the dental colleges. There should be more; there must be harmony between examining boards and the colleges, or there will be trouble that will retard that growth of scientific education that every lover of his profession is looking and working for.

It is time for the profession to let its voice be heard in this matter when thirteen states: Alabama, Colorado, Florida, Georgia, Maine, Massachusetts, Minnesota, Mississippi, New Jersey, New Hampshire, North Carolina, South Carolina and Virginia, have laws that do not recognize a diploma from any college, but all must stand an examination. Such a man as my friend, Dr. John E. Rich, who acquired a reputation before most of the men on the examining boards were born, could not go to and begin practice in any of the states named until he had been examined by a board. Again, what man is there who ten years or five years after graduation could stand a technical examination? I am sure I could not, and there are few who could. Is it right, is it just, to the old practitioner to be obliged to take up his school boy studies, if perchance he should wish to change his residence?

I believe in making a diploma mean something. Encourage and build up our colleges, make the diploma as hard to get as you please, put the student through as severe and hard an examination as you can make; but when he has once passed such an examination, give him his diploma and take him by the hand as a member of the profession who has earned a right to practice his calling in any part of these United States;and when this is done,it will give him a standing in other countries that is now not recognized, and will not be until we have shown confidence in our own work.

It was the work of dental colleges that suggested dental legislation, and called into existence dental examining boards. They are the very off-spring of our colleges, and like an ungrateful son, scorn their advice or teaching, thinking themselves wiser than their parents. A little more age may tend to give them wisdom and lead them to a better understanding of their true position and relation to the dental profession.

We need laws, but they should be framed with due thoughtfulness for the elevation, as well as the protection of the profession, and we must try and get men on examining boards who will have the mental and moral calibre to see beyond their own surroundings and the magnifying of their own importance. The National Board of Examiners, and the National Board of Faculties, have both been productive of great good, but their future usefulness will be curtailed, if not destroyed, unless harmony and unison of action be maintained between them. Let such legislation be had as will be for the support and encouragement of thorough education of those who propose to practice our profession. By the rejection of all diplomas a reflection is cast upon the examinations of students in our colleges. Who is there to review the work of the examining boards? Are they not quite as likely to err or be biased in their judgment if the work be alone conducted by them? A few of the states require as a prerequisite for examination a diploma, thus distinctly setting the examining board above the college, which looks to me like making the father subject to the whims of the son. We believe in colleges and college work, but I fear we shall see a deterioration in their work if their diplomas are not recognized. In fact, will not the student ignore them altogether and gain his knowledge by private pupilage with some dentist, who for a consideration will undertake to prepare him *for*, and even guarantee him the certificate *of*, an examining board through a friend on the board?

Will not the members of the Maryland State Society give this subject their thought and attention, so that harmony of action between your state board and colleges may serve as an example to other states?

We have no dental law in the District of Columbia yet, but we are working for one. A bill has passed the Senate and been favorably reported by the House, and we hope before this session of Congress has passed to have it enacted into law, and the graduates of your colleges will only be required to register, when they will be made welcome to practice our noble calling.

DISCUSSION OF DR. NOBLE'S PAPER.

After reading his paper, Dr. Noble said: Since I came to this meeting I see that you have recognized some of the difficulties in dental legislation and that you are taking steps for a new dental law in this state. The thing needs to be done in many of our states. I suppose you have read the paper by Dr. Sheppard of Boston. He is one of the State Examining Board for Massachusetts. He pledged himself as a member of the Massachusetts Board, to try to have the laws revised. But what we need is to have the general expression of the whole profession.

Dr. M. W. Foster.—The question of dental education and of harmony between the governing boards and faculties of the dental colleges is very important. How is it possible for a set of men, who are probably not always well fitted for their position, to decide justly? That these men should be entitled to say whether a graduate of any school in the country should be accepted or rejected is very discouraging to the dentists. There is certainly a chance that through political influence men will get on the governing boards of the states that are not just the proper men for the position. Therefore I can say that I am very glad Dr. Noble has taken the ground he has.

Dr. J. B. Rich.—Mr. President, it appears that this matter of examining boards is of vital importance to our profession. The manner in which governing boards are appointed is wrong in principle. The members of the board may be appointed through a good or a bad influence. Who can tell? A political influence is generally a bad influence. It seems to me to be an evil which calls for strong action at once. I think the members of this society ought to be agreed on this subject and, if they take the view which seems to me to be a plain common sense view, to act accordingly.

A member of the board of commissioners might be an inferior denti t, having secured his own appointment through improper influence, and yet he would have the right to say whether the graduates even of the best institution in the country shall have a right to practice in that city or not. I don't know but that it is really unconstitutional; that it is interfering with the rights of a citizen of the United States. The Supreme Court is coming around to this position.

If the graduates of some dental colleges are not sufficiently well trained, these colleges need to be brought up to the standard so that their diplomas shall be sufficient.

I think that it is only by the action of state societies that this matter can be remedied. Therefore I think that it is the duty of this society to take the initiatory movement. As this is the state in which the first dental college was established, I think that this society could well take the initiative.

Dr. Noble said that in this state the Examining Board say whether a college from which a dentist may come is reputable or not.

Dr. Rich.—I think this is more disgraceful yet.

Dr. Mills.—I believe that any law such as the above is against the rights of the citizens of the United States. Suppose a man enters a college which has a charter from the state. Any man has a right to appeal from the decision in regard to his diploma, if it is not accepted, and I think his appeal would be sustained by the Supreme Court. I think if a man once holds a diploma, that should be his passport. If a college is not reputable, it should be turned down by the state in which it is.

Dr. Rich.—I must apologize for taking the time of the society again. I was one of the first to help try to raise the profession from the slough in which it was situated. We have gone on and increased the numbers and raised the standard of our profession. Those who have succeeded, those who were first, have found a much easier path, their profession being recognized as a valuable one and one which is necessary to the community. But I think they have forgotten their duty. It is their duty to see that this movement which was started by us should go on continually. It is the duty of every man in this room to see that the elevation of his profession goes on all the time, that it receives no halt such as we have now met with. There should be some reorganization so that this examination should be done away with.

Dr. Noble.—Just one more thing. I believe that all the speakers have taken my side. I want here to call attention and emphasize the fact that this committee which you have appointed here will consider these things. I hope that they all will be carefully thought out and that the best legal talent will be employed in drawing up the bill to be presented to the Legislature.

---

### THE CONSERVATION OF EYE-SIGHT IN DENTAL OPERATIONS.

BY L. ASHLEY FAUGHT, D. D. S., PHILADELPHIA.

*Mr. President and Gentlemen:*

In choosing to speak to you to-day upon the "Conservation of Eye-sight I have not the slightest intention of delivering before your learned body, either a lecture upon the anatomy of the eye or upon the properties of light. With each of these topics and with their relations I am sure you are all sufficiently familiar. My sole object is to tell you of a few things which my experience has taught me will help the dental operator to preserve good eye-sight through an indefinite number of years.

We all know that the practice of dentistry perhaps more than other callings requires the keenest vision and a protracted use of the eyes. That they should be preserved in a healthy condition, under such conditions exercise is of the utmost importance. The abuses to which the eyes of the dentist are subjected may quite properly be classified into two divisions; those which occur during the hours in which he is engaged in his professional calling; and those which may occur while engaged in other occupations.

The first essential to the conservation of eye-sight during operating is that the light used shall be good. The meaning of this remark, if interpreted by an examination of what is accepted as good light, in the many dental offices situated as they are in such contrasting situations, is that derived from a window placed to command a more or less uninterrupted expanse of sky. Good light is however not essentially this, but is necessarily dependent upon fixed scientific principles. Its definition is not to be summed up in the wisdom of those who claim it to be plenty of light from a northern sky. To my mind the room chosen for operating should be lighted by a single aperture, for a complexity of light throws a complexity of shadows which will prove exceedingly trying to the eyes. This aperture should be so placed as to draw the light from as high a point as possible. Light derived from the lower half of an ordinary window, or from that portion of an aperture which is below an imaginary line placed a foot above the plane of the work is of little use and detrimental to the eyes. The light we work by comes from the strongest centre above, and all lower lights will prove cross-lights, conflicting with, rather than aiding true and healthful illumination. The light will be more even if the aperture be glazed by a single piece of glass, rather than by several panes.

The portions of sash supporting many pieces of glass make interruptions in light and throw shadows, and each separate pane too has a different power of refraction.

Whether the single aperture should face the north, south, east, or west, or any other point of the compass, is a question your essayist considers somewhat immaterial. The important factor in accepting any one of these facings is to remember that a *steady light of a definite uniform intensity* is what is desired; and if this exists not naturally, what is commanded must be moulded into this condition by suitable arrangements. It will fully repay each dentist to study to equalize his light; which will vary with the exposure, time of day, period of the year, serenity or obscurity of the sky, etc. This study each person must make for himself after having reached certain conditions which will be common to all. A wavering light is worse than one in which the intensity is lowered. Too little light is apt to produce near sighted condition of the eyes. Too much is the fault into which the majority fall—it over-stimulates the eyes. Beware of any light which passes through any material calculated to give it a change of color—such as calico, ground glass, etc.

The direction from which the light comes to the mouth of the patient is of great importance. It is universally agreed that for general art purposes that which comes from the left is best. Light from directly in front, pains the eye and inflicts an unconscious strain in the retina.

The less furniture in the operating room the better, and the walls should be finished in a neutral tint so that reflection in the operating room may be reduced to a minimum.

There can be no doubt in my mind of the efficacy of the use of a shade of black silk by the dentist, so that the pupil may be secured from interruption in its adjustment. The action of the eyes is perfect in proportion as their adjustment is perfect, and should be shaded from the intrusion of collateral rays.

The reported experience of others shows that the dentist should confine the use of his eyes during operating hours to natural light. Injury is sure to follow any attempt to prolong work by the use of artificial light. Some of my friends have tried the experiment, with the result that those who had not used glasses before were obliged to put them on, and those who wore them previously had to increase their magnifying power. These sad results occurring a few months after introducing the use of the stomatoscope into their offices.

If the patients are permitted to have the use of a hand mirror during operations, care is to be taken by the operator that they do not hold or place it in their laps, glass upwards. Invariably caution them to turn the back upwards, and save your eyes much irritation.

There is much else that I would like to say on this portion of my subject, but I always fear that I am bringing as it were coals to New Castle when I speak to professional men, so with your permission I will now make a few remarks upon the second division of the topic. The care of the eyes during the interval of office hours is quite necessary for it must be admitted to have a material effect in conserving them for dental operations.

The principle causes of injury during this period are from defective or excessive illumination, from excessive application, from unclean or impure air, from exposure to cold, from mechanical or chemical injury, from mental conditions, from unnatural conditions of the general system, and from want or misuse of spectacles. I do not intend to consider in detail all of these, but have mentioned them as leaders to the thoughts of others who may follow me in the discussion of my paper.

Care should be taken in passing from a dark to a brilliantly lighted place not to expose the eyes too suddenly. A similar strain is produced by the reflection from snow, and in Philadelphia from the enormous quantity of white marble and paint used in building construction.

The exposure of the eyes to bright sunlight on awakening in the morning has produced troublesome inflammation, and our beds should always be so placed that we may not sleep facing the morning sun unless the windows of the room are sufficiently covered by curtains.

Solar light is composed of different colors, which are ultimately resolvable into their primaries:—red, green and violet. These three are combined in the proportion of five parts of red, three of green, and eight of violet. The choice of an artificial light for reading should recognize this scientific statement.

Reading and working by two lights is to be depreciated. Sufficient illumination should be obtained from one, for with the two one portion of the retina is stimulated in excess of another.

Reading in direct sunlight, in the twilight, or in moving railway trains has laid the foundation of eye troubles for many people.

The smoking of tobacco is hurtful to the eyes if indulged in in a close railway carriage or in a room full of other smokers. I would also suggest the use of a mouth piece by those who habitually use cigars as it will tend to remove the smoke that comes from the end of the cigar to a greater distance from the eyes. For the same reason the smoke inhaled should be blown from the mouth in voiding it.

Now, gentlemen, in conclusion permit me to suggest that every dentist owes it to himself and to his patients to consult his oculist at least twice in each year. These consultations are best conducted, say, in July and in October. They are seasons at the conclusion and at the commencement of his winter's work, and the results of one examination may correct the other. After the eyes have had the rest of the summer, the October examination may save the use of the glasses which the July examination may have indicated, and yet the July examination may show sufficient overstrain from the winter's work to need immediate relief or correction.

The condition of the eye-sight your essayist has often found to be a good barometer of the condition of his health, and this he believes to be true of many others, and attention to the eyes may direct repairs to the nervous system in general.

DISCUSSION.

Dr. C. C. Harris.—While I do not want to be conflicting, I will say that in my early years I took a course in medicine. I found, according to authorities, that after a certain age there was no danger of near-sightedness. According to these teachings, it becomes impossible for a person, with normal eye-sight, to become near-sighted after the age of 25 or 30 years.

Dr. B. Holly Smith.—I have been interested in the paper read. On account of my eyes I have been obliged to have a person read to me after a hard day's work. The different things the essayist has suggested have been very valuable suggestions to me.

Dr. Harris.—I hope no one will think that I didn't properly appreciate the doctor's paper. I simply wanted to state a fact which was called to my attention. I appreciate the paper very much.

Dr. H. B. Noble.—I think such a paper ought not to pass without laying emphasis on the important things mentioned there. What the essayist has brought out in reference to the selection of our light is very important. A northern light is a steady light but it is certainly a weaker light. For some time I used a northern light, but I don't think it is the best now. The light, I think, should come from one window and should be as clear and distinct as possible. I have found that light from the lower sash is not desirable. It is brought out in the paper that we should not have anything in the room to reflect the light to our eyes.

What he said about consulting an oculist was a new idea to me. I think hereafter I will give this regular attention. I recognize that the eye, as the doctor said, is the thermometer of our regular health.

I certainly desire to thank the doctor for his paper. It is one which will do us all good I am sure.

Dr. R. B. Winder.—I did not hear the paper, but there are some things which are important. The abuse of students' eyes over microscopes is to be avoided. I have the opinion of some of the most distinguished oculists of this country upon this point. For this reason I have never advocated the use of microscopes in the dental colleges. The dentist's hands and eyes are very important to him, and must be preserved for the successful prosecution of his business.

Dr. J. B. Rich.—I don't know that any more important paper could be presented than this which emphasizes the care of the eyes. Any attempt to increase the power of the light is dangerous to the dentist. I once thought of inventing an apparatus for increasing the power of the light on dark days, but I came to the conclusion that this would be injurious.

I think south light is the best because of the variation in it.

I beg leave to differ from Dr. Winder on the use of the microscope. From my own experience and from the experience of others I don't think the eye is ever injured by the use of the microscope. I have been an expert microscopist for a long time, and I think you will find very few persons having as good eyes as I have, considering that I am 81 years old. I should be sorry to see the mass of my profession shut out from the use of the microscope.

Early in my professional life, when my eyes were strong, I adopted the practice of using glasses while performing my dental operations. Perhaps I owe my good eye-sight to this. Just as engravers need to assist their eye-sight in this way, so do dentists.

Dr. A. J. Volck.—I am fully in accord with what has just been said in regard to the use of the microscope. I think the dentists will one after another see the necessity of taking up the microscope, especially in the study of diseased teeth.

I am pretty old myself. I have worked pretty hard at my profession. Have worked at things which require very good eye-sight. When I have had to take up fine work I have used glasses. I have just as good eye-sight now as when I was ten years old. What we need to do is to take good care of ourselves, especially of our digestive organs.

Dr. Rich.—I mentioned the microscope to you dentists. A few hints to you who are studying with them: Be careful to use as low powers as you can in the work you have to do; use as low an illumination as possible; also try to procure Ranney's moderator which takes away the red rays from the light. It is the red rays which are injurious to the eyes. Always use as white light as possible.

Dr. Winder.— In my remarks in regard to the microscope I was not speaking of men of especially strong constitution but of men in general. If a person here has as good eye-sight as when he was ten years old, I don't think the microscope or any thing else would injure his eyes. I was speaking of the ordinary person. I think Dr. Rich has been a man of the strongest constitution in every particular. I am speaking of the cases where the use of the microscope injures the eye, and I think in the case of the ordinary person it does injure it.

Dr. Rich.—In my remarks of course I did not mean the abuse of the microscope, but I mean that with the proper use of the microscope I have never known of a single instance where the eye has not been benefited.

Dr. Lewis Buffett.—I commenced the use of the microscope about 20 years ago. I began spending three hours twice a week and my eyes failed me. I had to stop. It is hard to say what is meant by the proper use of the microscope. Some persons can use the microscope day after day without any bad result. A proper use of the eye will strengthen it. The dentist who uses his eyes rightly will find them strengthened. But the proper use differs with the individual.

Dr. Bonwill.—This is a very important subject. I can only give my own experience. For a long time I have used my eyes severely, often working from seven o'clock in the morning until one o'clock the next morning, yet my eyes are in good condition. Recently I found that I needed weaker glasses than I had been using. A man can excessively use his eyes if he keeps the rest of his body in good condition.

There is an advantage in using double glasses. You can readily look at either near or distant objects. You don't really know that you have glasses on.

I have found that a combination of northern light with east and west lights has been best for me. I always stand with my back to the light. Another point is to wear a shade over your eyes. In taking care of your bodily health, place your stomach first of all. Take care of what you eat.

Dr. Faught.—-My experience bears out what I said in regard to the physical condition influencing the eyes. What I have given in the paper I have given as a result of practical study upon my own eyes. The suggestion of keeping the back to the light is important.

Dr. Harris in his criticism fell into a little error which was natural. I did not say that too little light would produce near-sightedness. I said that it was apt to produce the *condition* of near-sightedness.

In regard to the microscope I would say that I have used it a great deal, and I heartily agree with what has been said in its favor. When working with it you want a minimum amount of light and the use of color glasses. With proper care we can use the microscope without detriment. Sometimes you may find one eye falling below the normal and then coming back again.

In regard to my eyes the oculist says that each year my eye-sight is improving.

Dr. Winder.—I don't want to be misunderstood. I don't condemn the use of the microscope. What I condemn is the indiscriminate use of the microscope in the dental colleges. The indiscriminate use of the microscope in the colleges, requiring the same amount of work from all, regardless of the strength of their eyes, is wrong.

---

ARTICLE II.

